# Dynamic Interaction of TTDA with TFIIH Is Stabilized by Nucleotide Excision Repair in Living Cells

**DOI:** 10.1371/journal.pbio.0040156

**Published:** 2006-05-09

**Authors:** Giuseppina Giglia-Mari, Catherine Miquel, Arjan F Theil, Pierre-Olivier Mari, Deborah Hoogstraten, Jessica M. Y Ng, Christoffel Dinant, Jan H. J Hoeijmakers, Wim Vermeulen

**Affiliations:** **1**Department of Cell Biology and Genetics, Medical Genetics Center, Erasmus Medical Center, Rotterdam, Netherlands; Lawrence Livermore National LaboratoryUnited States of America

## Abstract

Transcription/repair factor IIH (TFIIH) is essential for RNA polymerase II transcription and nucleotide excision repair (NER). This multi-subunit complex consists of ten polypeptides, including the recently identified small 8-kDa trichothiodystrophy group A (TTDA)/ hTFB5 protein. Patients belonging to the rare neurodevelopmental repair syndrome TTD-A carry inactivating mutations in the *TTDA/hTFB5* gene. One of these mutations completely inactivates the protein, whereas other TFIIH genes only tolerate point mutations that do not compromise the essential role in transcription. Nevertheless, the severe NER-deficiency in TTD-A suggests that the TTDA protein is critical for repair. Using a fluorescently tagged and biologically active version of TTDA, we have investigated the involvement of TTDA in repair and transcription in living cells. Under non-challenging conditions, TTDA is present in two distinct kinetic pools: one bound to TFIIH, and a free fraction that shuttles between the cytoplasm and nucleus. After induction of NER-specific DNA lesions, the equilibrium between these two pools dramatically shifts towards a more stable association of TTDA to TFIIH. Modulating transcriptional activity in cells did not induce a similar shift in this equilibrium. Surprisingly, DNA conformations that only provoke an abortive-type of NER reaction do not result into a more stable incorporation of TTDA into TFIIH. These findings identify TTDA as the first TFIIH subunit with a primarily NER-dedicated role in vivo and indicate that its interaction with TFIIH reflects productive NER.

## Abstract 2

Transcription/repair factor IIH (TFIIH) is a multi-subunit protein complex essential for RNA polymerase II transcription and nucleotide excision repair (NER). The authors show that the TTDA subunit is associated with TFIIH specifically during NER.

## Introduction

DNA-damaging agents constantly challenge the integrity of DNA. A network of distinct DNA-repair systems collectively removes most injuries and safeguards the stability of the genome [ 1]. Nucleotide excision repair (NER) is a DNA repair mechanism capable of removing different structurally unrelated DNA helix-distorting lesions, including ultraviolet light (UV)-induced lesions and bulky chemical adducts. NER requires the concerted action of ˜25 proteins in a coordinated multi-step process [ 2]. After initial damage recognition, the DNA helix is opened by the bi-directional helicase function of TFIIH. Subsequently, four proteins (xeroderma pigmentosum complementation group G protein [XPG], xeroderma pigmentosum complementation group A protein [XPA], replication protein A, and excision repair cross complementing group 1 protein [ERCC1] in complex with xeroderma pigmentosum group F [XPF]) are recruited to stabilize the open intermediate, verify the damage, and incise the damaged strand 3′ and 5′ at some distance from the injury [ 3]. Finally, the resulting gap is filled by repair replication and sealed by ligation. Within NER, two lesion recognition pathways are operational: transcription-coupled NER (TC-NER) and global genome NER (GG-NER) [ 4]. TC-NER is dedicated to lesions that block RNA polymerase II elongation. Within TC-NER, the stalled RNA polymerase likely first detects lesions whereas also the Cockayne syndrome A and B proteins play roles in the early steps of this process. The GG-NER-specific complexes (xeroderma pigmentosum complementation group C [XPC] in complex with the human homologue of Rad23 [hHR23B/A], and xeroderma pigmentosum complementation group E [XPE/DDB2] in complex with the UV-damaged DNA binding protein 1, [UV-DDB1]) recognize lesions at any position in the genome [ 5]. GG-NER mainly protects against damage-induced mutagenesis and can thus be considered as a cancer-preventing process, whereas TC-NER primarily promotes cellular survival, and therefore may prevent aging [ 6]. Hereditary NER deficiency is associated with severe clinical features as presented by three photo-sensitive disorders: the cancer-prone syndrome xeroderma pigmentosum (XP) and the neurodevelopmental conditions Cockayne syndrome and trichothiodystrophy (TTD)[ 7]. TTD is a premature aging syndrome, with the hallmark feature of brittle hair and nails, ichthyosis, and progressive mental and physical retardation also seen in Cockayne syndrome. Within photo-sensitive TTD, three TFIIH coding genes are implicated: xeroderma pigmentosum complementation group B (XPB) [ 8], xeroderma pigmentosum complementation group D (XPD) [ 9, 10], and the newly identified protein termed “TTD group A” (TTDA) [ 11, 12].

Besides GG-NER and TC-NER, TFIIH is also engaged in RNA polymerase II transcription initiation, RNA polymerase I transcription, activated transcription, and cell cycle regulation [ 13– 20]. TFIIH consists of ten subunits, five of which (XPB, p62, p52, p44, and p34) form a tight core-complex, and the trimeric cyclin activating kinase-subcomplex (CDK7, MAT1, and cyclin H) is linked to the core via the XPD protein [ 21]. The recently identified 8-kDa TTDA protein [ 11, 12] connects to the core via interactions with p52 and XPD [29]. TFIIH harbors different enzymatic activities: two DNA-dependent ATPases, XPB and XPD, required for the helicase function [ 22, 23], a protein kinase displayed by CDK7 [ 24], and the recently uncovered ubiquitin ligase activity of p44 [ 25]. Currently, the functions or possible enzymatic properties of the other TFIIH subunits including that of TTDA remain enigmatic.

Cells from patients with TTD-A have reduced steady-state levels of TFIIH, due to mutated *TTDA* gene [ 26], suggesting that an important role of TTDA is to stabilize the entire TFIIH complex. The most striking feature of TTD-A cells is their reduced DNA repair activity. Intriguingly, the identified mutations, within the *TTDA* gene of three non-related patients with TTD-A, lead either to the complete absence of the protein (mutation on the first ATG), or to non-functional truncated peptides, making TTDA the first TFIIH subunit for which a complete absence is compatible with life [ 12]. Despite these rather diverse mutations, a surprisingly similar expression of the clinical features is observed amongst the patients.

In order to investigate the participation of TTDA in DNA repair and transcription, we measured the differences in mobility of TTDA during repair and transcription in living cells by confocal imaging of a functional green fluorescent protein (GFP)-tagged TTDA (TTDA-GFP) expressed in TTDA-deficient transformed fibroblasts. To compare the mobility of TTDA with others TFIIH subunits, we also measured kinetic parameters of XPB-GFP and XPD-GFP in each of these processes.

## Results

### Expression and Functionality of TTDA-GFP and XPD-GFP

**Figure 1:**
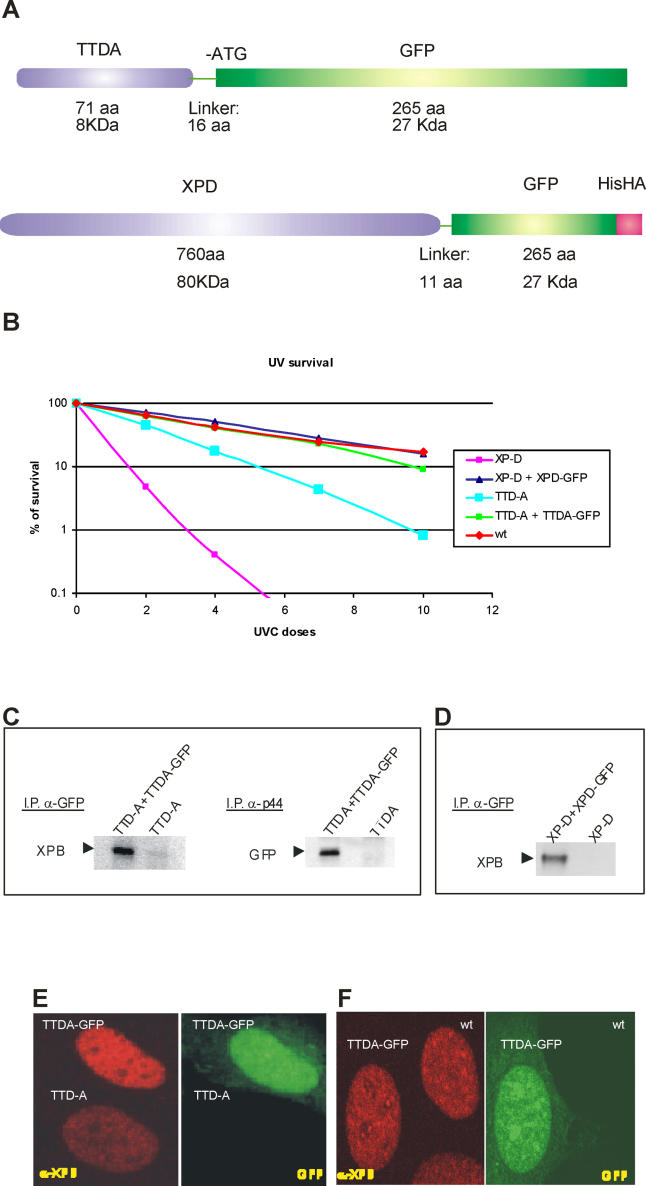
Constructs and Functionality Assays (A) Scheme of the TTD-A-GFP and XPD-GFP fusion proteins. (B) UV survival using wt VH10 cells (red diamonds), TTD1BRSV (TTD-A) cells (light blue squares), XP6BESV (XP-D) cells (pink squares), TTD1BRSV-expressing TTDA-GFP cells (green squares), and XP6BESV-expressing XPD-GFP cells (blue triangles). The percentage of surviving cells is plotted against the applied UV-C dose (J/m2). (C) Immunoprecipitation: Left panel shows that the XPB TFIIH subunit (by monoclonal anti-XPB detection) co-precipitates with anti-GFP from extracts of TTDA-GFP-expressing fibroblasts (lane 1) and not from TTD1BRSV (TTD-A) fibroblast whole-cell extracts (lane 2). Right panel shows that TTDA-GFP (detected with anti-GFP monoclonal) co-precipitates with the core TFIIH component p44 from extracts from TTDA-GFP-expressing fibroblasts (lane 1) and not in whole-cell extracts from TTD1BRSV (TTD-A) fibroblasts (lane 2). (D) Immunoprecipitation using polyclonal anti-GFP. Also the XPB TFIIH subunit co-precipitated with XPD-GFP (using anti-GFP) in extracts from XPD-GFP-expressing fibroblasts (lane 1), but not from XP6BESV (XP-D) fibroblasts (lane 2). (E) Immunofluorescence of a mixed population of TTD-A cells (label 2) and TTDA-GFP-expressing TTD-A cells. Cells expressing TTDA-GFP (right panel) showed an increased level of XPB (left panel), compared to TTD-A cells. (F) Immunofluorescence of a mixed population of VH10 (wt) cells and TTDA-GFP-expressing TTD-A cells. Cells expressing TTDA-GFP (right panel) exhibit a similar expression level of XPB (left panel) as wt cells.

To investigate the localization, behavior and dynamic properties of TTDA in the most relevant biologically context—the living cell, and under different conditions, such as transcriptional interference and introduction of DNA lesions, we have generated a GFP-tagged TTD A ( Figure 1). To compare the behavior of TTDA-GFP with other TFIIH components, we also produced an XPD-GFP fusion ( Figure 1A) and used a cell line stably expressing XPB-GFP [ 14]. The cDNAs encoding TTDA-GFP and XPD-GFP were expressed in TTD-A and XP-D SV40-immortalized human fibroblasts, respectively. Functionality of the fusion proteins was tested, in stably expressing clones, by determining the UV sensitivity ( Figure 1B) and recovery of RNA synthesis after UV irradiation (unpublished data). Both TTDA-GFP and XPD-GFP restored both NER-deficient parameters in TTD-A and XP-D cells, respectively, showing that both fluorescently marked proteins are completely functional in repair.

Immunoprecipitation assays showed that the TFIIH core factor, XPB, co-precipitates with both fusion proteins, using anti-GFP cross-linked sepharose beads and extracts of TTDA-GFP ( Figure 1C, left panel) and XPD-GFP-expressing cells ( Figure 1D). In addition, TTDA-GFP co-precipitated with anti-p44 ( Figure 1C, right panel). These results showed that XPD-GFP and TTDA-GFP were incorporated into TFIIH, despite the relatively large GFP tag, particularly considering the very small size of the TTDA subunit. Moreover, the severely reduced amount of TFIIH in TTD-A cells [ 26] was restored to wild-type (wt) levels ( Figure 1E) by TTDA-GFP expression, as was previously observed for Hemagglutinin peptide-tagged TTDA (TTDA–HA)-expressing cells [ 12]. Interestingly, the stable expression of TTDA-GFP does not induce higher levels of TFIIH than in wt cells ( Figure 1F). We conclude from these data that we have generated two cell lines that stably express biological active fluorescently tagged subunits of TFIIH, which are bona fide tools to study the spatiotemporal distribution and protein dynamics of TFIIH during repair and transcription.

### Cellular Localization of TTDA-GFP and XPD-GFP

**Figure 2:**
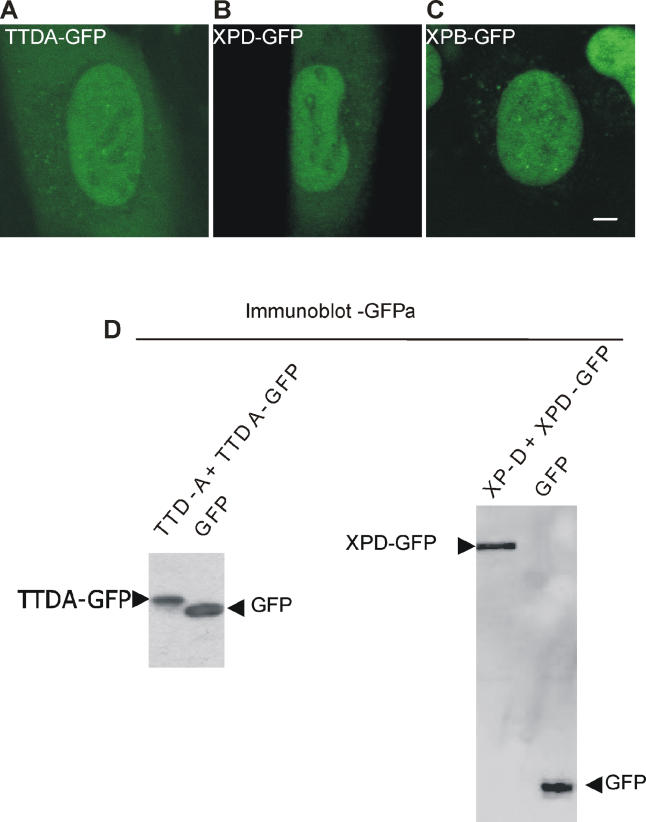
Localization of TTDA-GFP and XPD-GFP (A) Confocal image of a TTDA-GFP-expressing cell. (B) Confocal image of a XPD-GFP-expressing cell. (C) Confocal image of a XPB-GFP-expressing cell. (D) Immunoblot probed with anti-GFP monoclonal antibody of TTD1BRSV (TTD-A) fibroblasts stably expressing TTDA-GFP (lane 1), XP6BESV (XP-D) transformed fibroblasts stably expressing XPD-GFP (lane 3), and MRC5SV (wt) transformed fibroblasts expressing free GFP (lane 2 and 4).

High-resolution confocal imaging ( Figure 2) of TTDA-GFP- and XPD-GFP-expressing cells revealed a strikingly similar localization. Both present a cytoplasmic and nuclear localization ( Figure 2A and 2B), sharply contrasting to XPB-GFP that is strictly nuclear [ 14] ( Figure 2C). Cytoplasmic expression of XPD-GFP is not unexpected since it was previously observed [ 27] and endogenously expressed XPD was found in both cytoplasmic and nuclear fractions after subcellular fractionation (unpublished data). Cytoplasmic expression is not due to alternative translation starts or fusion protein breakdown products, because immunoblot analysis using an antibody against GFP revealed the presence of only intact XPD-GFP and TTDA-GFP ( Figure 2D). Previous immunofluorescent analysis revealed that TTDA-HA was predominantly localized in the nucleus [ 12]; however, this technique includes permeabilization, which will wash out the cytoplasmic pool of TTDA-HA. In addition, both TTDA-GFP and XPD-GFP accumulate in nucleolar foci, previously also observed in fixed TTDA-HA-expressing fibroblasts [ 12] and in living cells expressing XPB-GFP [ 14].

### Mobility of TTDA-GFP and XPD-GFP in Different Cellular Compartments

**Figure 3:**
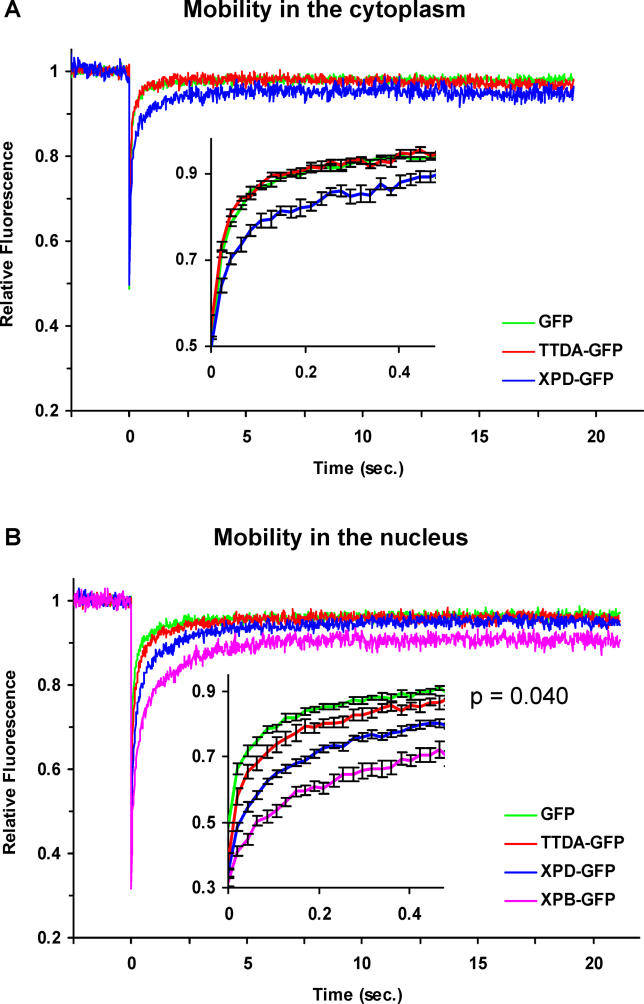
Mobility of TTDA-GFP and XPD-GFP in the Cytoplasm and in the Nucleus Determined by FRAP (A) FRAP analysis of XPD-GFP (blue line), TTDA-GFP (red line), and free GFP (green line) residing in the cytoplasm. Inset shows increased time resolution of the curves with error bars. (B) FRAP analysis of XPB-GFP (pink line), XPD-GFP (blue line), TTDA-GFP (red line), and free GFP (green line) in the nucleus. Relative fluorescence (fluorescence post-bleach divided by fluorescence pre-bleach) plotted against time in seconds. Inset shows increased time resolution of the curves with error bars. The *p*-value has been calculated for GFP and TTDA-GFP datasets.

The cytoplasmic localization of both XPD-GFP and TTDA-GFP argues for a possible assembly of TFIIH-related subcomplexes within the cytoplasm. In order to investigate this, we measured the mobility of TTDA-GFP, XPB-GFP, XPD-GFP, and GFP in both the cytoplasm and the nucleus by photo-bleaching experiments. We applied a tailor-made variant of fluorescence recovery after photo-bleaching (FRAP) [ 28] in which fluorescent molecules are photo-bleached in a small strip by a high intensity laser pulse, the subsequent fluorescence recovery in time is monitored and is a measure for the protein mobility ( Figure 3).

Within the cytoplasm ( Figure 3A), the mobility curve of TTDA-GFP virtually overlaps with that of non-tagged GFP and both show a significant faster fluorescence recovery than cytoplasmic XPD-GFP. These results suggest that the majority of TTDA-GFP and XPD-GFP do not interact with each other in the cytoplasm of living cells, despite the fact that these proteins do interact in the context of TFIIH [ 29]. Furthermore, the similar mobility rate of GFP and TTDA-GFP argued for a mobility of TTDA-GFP that is mainly determined by passive diffusion on its own, very comparable to GFP [ 30]. In the nucleus ( Figure 3B), TTDA-GFP and XPD-GFP exhibit a slower mobility than in the cytosol ( Figure 3A and 3B). In addition, the overall nuclear mobility of TTDA-GFP is slower than that of free GFP. Surprisingly however, even in the nucleus both XPD-GFP and TTDA-GFP move much faster than XPB-GFP ( Figure 3B). The relative slow mobility of XPB-GFP was previously explained by its incorporation into the large TFIIH complex with a predicted molecular size of ˜500 kDa which is even more retarded by its ”stop and go” interactions with the transcriptional machinery at promoter sites [ 14]. Immunoprecipitation experiments showed however that both TTDA-GFP and XPD-GFP are also associated with TFIIH ( Figure 1C and 1D). A possible explanation for this unexpected high motility and apparent discrepancy is that the determined mobility reflects the mean of two (or more) components: a slow one, representing the fraction of molecules incorporated in TFIIH, and a fast one, representing the unbound pool of TTDA-GFP and XPD-GFP molecules.

**Figure 4:**
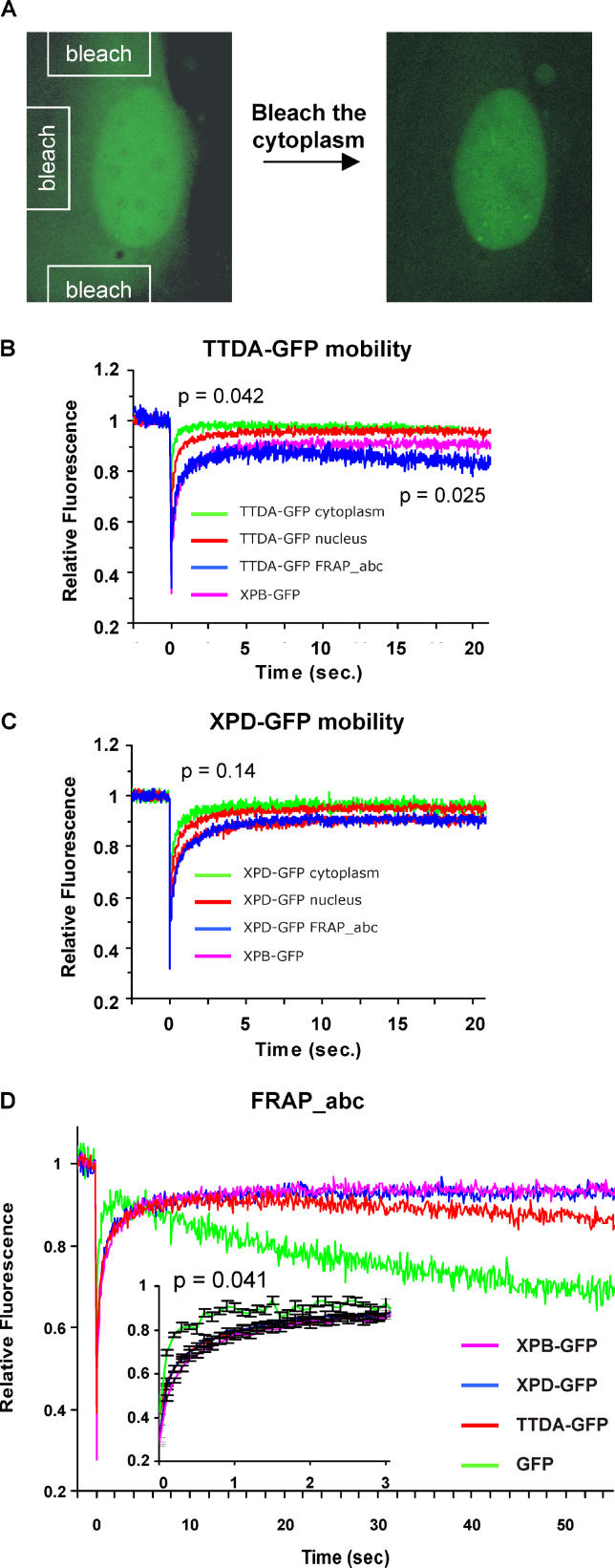
FRAP_abc (A) TTDA-GFP-expressing fibroblast without treatment (left panel) and after (right panel) applying several high laser pulses in the cytoplasmic compartment (see Materials and Methods for details). (B) TTDA-GFP mobility in the cytoplasm (green line), in the nucleus without bleaching the cytoplasm (red line), in the nucleus after bleaching the cytoplasmic fraction (blue line), and XPB-GFP mobility in the nucleus (pink line). The *p*-value has been calculated for cytoplasmic and nuclear mobility curves of TTDA-GFP. (C) XPD-GFP mobility in the cytoplasm (green line), in the nucleus without bleaching the cytoplasm (red line), in the nucleus after bleaching the cytoplasmic fraction (blue line), and XPB-GFP mobility in the nucleus (pink line). The *p*-value has been calculated for cytoplasmic and nuclear mobility curves of XPD-GFP. (D) FRAP_abc on XPB-GFP (pink line), XPD-GFP (blue line), TTDA-GFP (red line), and free GFP (green line). Inset shows increased time resolution of the curves with error bars.

In order to eliminate the fluorescence of this non-incorporated fraction, we modified the FRAP procedure by pre-bleaching the cytoplasmic fraction (see Materials and Methods and Figure 4). Assuming protein exchange between the nucleoplasm and the cytoplasm this procedure will photo-bleach both cytosolic and nuclear free fractions ( Figure 4A). Subsequent FRAP analysis in the nucleus, which for simplicity we have called FRAP after bleaching the cytoplasm (FRAP_abc) allowed us to measure the mobility of possibly incorporated TTDA-GFP and XPD-GFP into nuclear complexes. When we applied FRAP_abc, we observed a striking reduction of TTDA-GFP ( Figure 4B) and XPD-GFP ( Figure 4C) mobility that was very similar to XPB-GFP assayed in parallel and significantly slower than GFP ( Figure 4D). However, only the initial recovery of TTDA-GFP (i.e., the early time points after photo-bleaching) was similar to XPB-GFP. At later time points a surprising decline of the initial recovery of TTDA-GFP was observed, whereas the XPD-GFP recovery-curve remained stable and indistinguishable from XPB-GFP ( Figure 4D). The remarkable downward slope of the recovery curve was also detected, though much more pronounced, with free GFP ( Figure 4D) and only occurred after FRAP_abc (when cytoplasmic GFP molecules were bleached), implying that exchange occurred of bleached and non-bleached GFP molecules with the cytoplasm through the nuclear pores and vice-versa. This is consistent with increased fluorescence in the bleached cytoplasm (unpublished data). The reciprocal experiment i.e. bleaching of the nuclear pool of TTDA-GFP ( Figure S1), confirmed that the mobility of the cytoplasmic pool of TTDA-GFP mainly represents freely mobile, non-complexed TTDA-GFP. The similar early fluorescence recovery profile of all TFIIH components tested, suggested that the initial recovery is mainly determined by the molecules moving through the nuclear space. As argued above the subsequent loss of fluorescence at later time points can be explained by exchange through the nuclear membrane. This latter process has a slower kinetics due to spatial restrictions (change of diffusion through a nuclear pore), explaining its minute contribution to the relatively fast initial recovery. Because this fluorescence decline did not occur with the XPB and XPD TFIIH components, but only with TTDA-GFP, we hypothesized that TTDA is not stably associated to TFIIH. This implies that in these non-challenged cells, equilibrium exists between two kinetic pools of TTDA-GFP molecules, i.e. a free fraction and a TFIIH-bound pool. The fact that free GFP exchanges much faster than TTDA-GFP, though both are almost identical in size, supports the idea that TTDA-GFP exchange over the nuclear membrane is retarded by its incorporation into TFIIH.

### Mobility of TTDA-GFP after UV Irradiation

**Figure 5:**
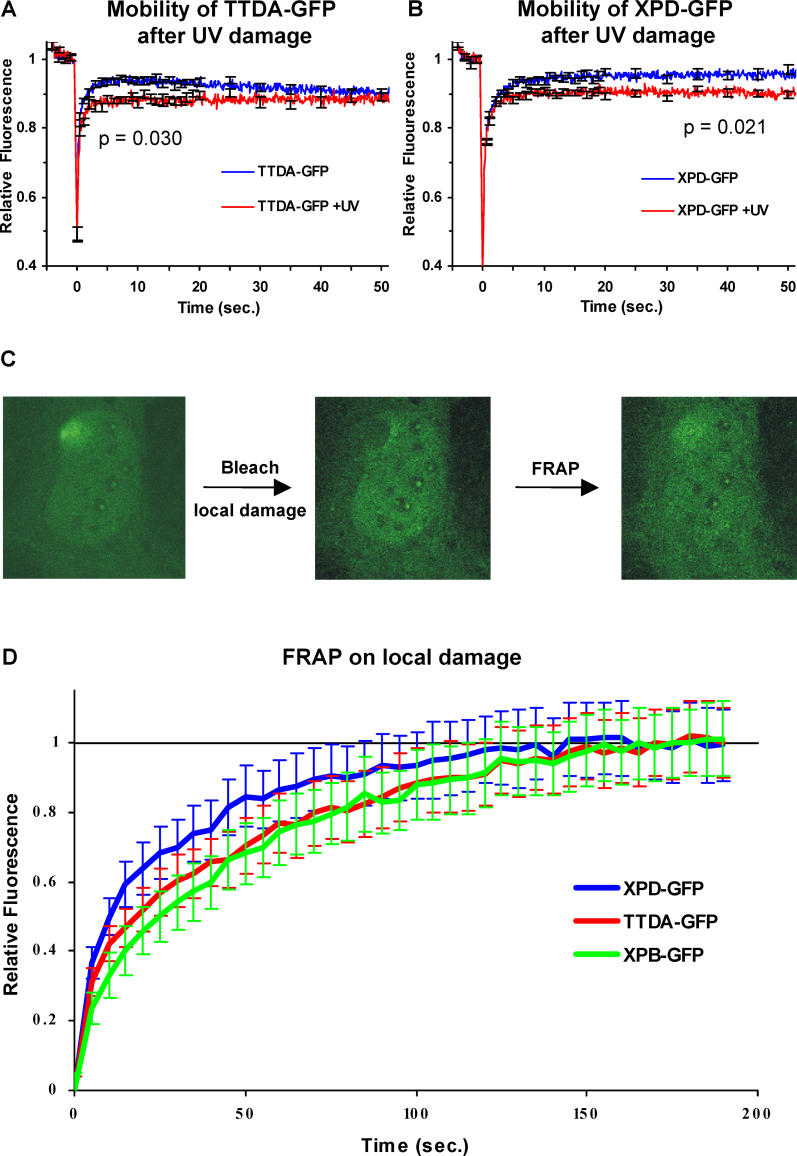
Mobility of TTDA-GFP and XPD-GFP after UV Irradiation (A) FRAP_abc of TTDA-GFP expressing cells untreated (blue line) and treated with 8J/m2 UV-C (red line). Error bars are included in the curves, and the *p*-value has been calculated for the two distinct datasets. (B) FRAP_abc of XPD-GFP-expressing cells untreated (blue line) and treated with 8J/m2 UV-C (red line). For each line at least 20 different cells were measured. Error bars are included in the curves, and the *p*-value has been calculated for the two distinct datasets. (C) Example of FRAP on local damage. A TTDA-GFP-expressing cell (left panel) containing a locally inflicted UV-damaged spot (shown by the white arrow). The locally damaged area is bleached by applying a high-pulse laser beam (middle panel), and the subsequent recovery of fluorescence is followed in time (right panel). (D) Curves of FRAP on local damage of TTDA-GFP- (red line), XPD-GFP- (blue line), and XPB-GFP-(green line) expressing cells. FLD, fluorescence measured on local damage; FNLD, fluorescence measured on untreated areas. Error bars are included in the curves.

To investigate whether the presence of DNA damage influences the equilibrium between the various TTDA-GFP pools, we compared the dynamic behavior of TTDA-GFP before and after UV irradiation (measured between 5 to 30 min after exposure) ( Figure 5). FRAP_abc revealed that after UV both XPD-GFP and TTDA-GFP exhibit an incomplete recovery, indicative for a relatively long immobilization ( Figure 5A and 5B), previously explained by the physical participation in the repair reaction [ 14, 28, 31, 32]. Surprisingly, the downward slope, as observed in the mobility curve of untreated TTDA-GFP-expressing cells, is significantly reduced when cells are treated with UV. This observation can be explained as a reduction of the pool size of free TTDA-GFP, thus attenuating the effect of exchange with the cytoplasmic bleached pool. This phenomenon argues for a more stable integration of TTDA into TFIIH when the complex is engaged in the NER reaction. In order to show that the stabilized association of TTDA to TFIIH is derived from actual participation in NER, we reduced the XPC concentration by RNA interference. Because XPC is the initiator of GG-NER, its depletion would prevent the formation of NER complexes on damaged DNA and thus should not induce damage-dependent immobilization of subsequent NER factors. When we transfected our cells that stably express TTDA-GFP with a mixture of two small interference RNA constructs against XPC ( Figure S2), a reduction of the immobile fraction can be observed in irradiated TTDA-GFP-expressing cells ( Figure S2C), showing that indeed the integration of TTDA in TFIIH is dependent on NER.

We further explored the participation of the three TFIIH components in NER by determining the binding time of each of the components when actively engaged in NER. To that aim, we applied FRAP on locally UV-damaged cells ( Figure 5C), as was previously determined for XPB-GFP [ 14]. As shown in Figure 5D, both TTDA-GFP and XPD-GFP have a similar average turnover time as XPB-GFP on locally damaged areas (time at which the equilibrium between damaged area and the rest of the nucleoplasm is re-established). These data argue for a similar dynamic behavior of the three measured TFIIH components within NER and furthermore imply that the association between TTDA-GFP and TFIIH is stronger or longer after UV damage.

### Mobility of TTDA-GFP after Transcription Inhibition

**Figure 6:**
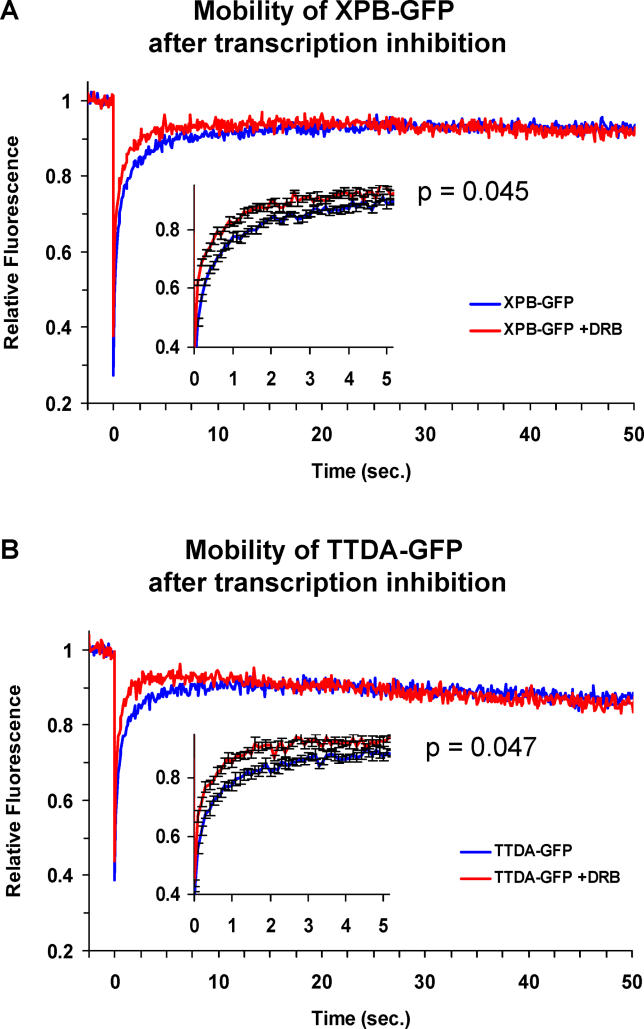
Mobility of XPB-GFP, TTDA-GFP after Transcription Inhibition (A) FRAP_abc of XPB-GFP-expressing cells untreated (blue line) and treated with dRB (see Materials and Methods) (red line). Inset shows increased time resolution of the curves with error bars. The *p*-value has been calculated for the two distinct datasets. (B) FRAP_abc of TTDA-GFP-expressing cells untreated (blue line) and treated with dRB (red line). Inset shows increased time resolution of the curves with error bars. The *p*-value has been calculated for the two distinct datasets.

This tighter association of TTDA-GFP to TFIIH during repair can be explained in two ways: TTDA is more tightly anchored to TFIIH when (non-specifically) bound to DNA or TTDA is indeed a NER-specific subunit of TFIIH docking with higher affinity to the complex when actively repairing. A standard not NER-related DNA interaction of TFIIH is of course its transient interaction with promoter sequences during transcription initiation. Varying the transcriptional competence of the cells would thus also affect the equilibrium of TFIIH-bound versus free TTDA. To verify this hypothesis, we measure the mobility of TTDA-GFP during inhibition of transcription by DRB (5,6-dichloro-1-beta-D-ribofuranosylbenzimidazole), known to increase overall mobility of XPB-GFP by the reduced number of interactions with transcription initiation complexes [ 14]. As shown in Figure 6, both TTDA-GFP and XPB-GFP exhibit a similar sensitivity towards DRB in terms of the effect that this drug has on mobility. However, the rate of exchange of molecules over the nuclear pores is not very much changed as could be inferred from the similar fluorescence decline after the initial recovery, suggesting that the equilibrium is not significantly affected by transcriptional interference. Thus, TFIIH binding per se does not affect TTDA pool equilibrium and suggests a more stable association of TTDA to TFIIH, when TFIIH is bound specifically in a NER-dependent reaction.

### Accumulation of TTDA-GFP on Actinomycin D/488 nm Laser-Induced Damage

**Figure 7:**
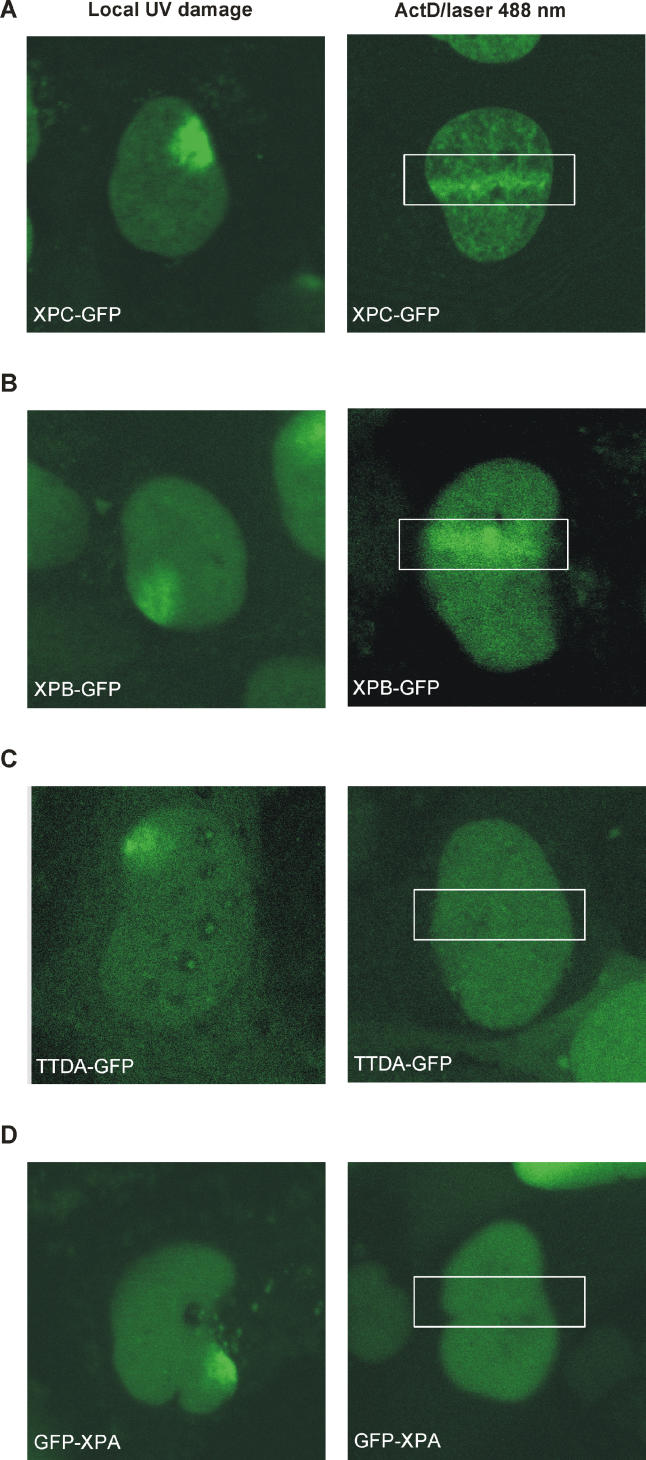
Comparison of Accumulation of NER Proteins on Local UV Damage and on Localized ActD-Photosensitized Laser Damage (A–D) Left panel, local DNA damage infliction by UV-C irradiation through a micro-porous filter. Right panel, local laser-induced (488 nm) DNA lesions by photo-sensitization of ActD. (A) XPC-GFP-expressing cells showing accumulation at local UV-damaged area (left panel) and laser induced ActD-damaged area (right panel). (B) XPB-GFP expressing cells showing accumulation at local UV-damaged area (left panel) and laser induced ActD-damaged area (right panel). (C) TTDA-GFP expressing cells showing only accumulation on local UV-damaged area (left panel) but not on laser induced ActD-damaged area (right panel); drawn rectangle corresponds with the irradiated area. (D) GFP-XPA expressing cells showing only accumulation on local UV-damaged area (left panel) but not on laser induced ActD-damaged area (right panel); drawn rectangle corresponds with the irradiated area.

Next we tested the effect of actinomycin D (ActD), classically used as a transcription elongation inhibitor but also known to interfere with other DNA transactions [ 33– 35]. In contrast to transcriptional inhibition by DRB, ActD induces an apparent increase of immobilized XPB-GFP (unpublished data), similar to previous observations with RNA polymerase II [ 36] and the TC-NER specific factor CSB [ 32]. Upon closer inspection of XPB-GFP mobility after ActD treatment and following fluorescence recovery over a longer time we noticed a surprising increase of fluorescence in the bleached strip, reaching even a higher level than in the non-bleached areas of the nucleus, arguing for locally accumulated TFIIH molecules in the region that was bleached. It is known that photo-activation of intercalated ActD by exposure to blue light (>400 nm), induces a variety of DNA lesions, such as single-strand breaks, oxidative damages, and probably also bulky lesions [ 37]. It is therefore likely that treatment of cells with ActD and subsequent illumination by 488 nm (i.e. the excitation wavelength of GFP) causes DNA lesions that recruit NER factors. To test this possibility in addition to TTDA-GFP- and XPB-GFP-expressing cells, we also treated XPC-GFP- and GFP-XPA-expressing cells with ActD and locally photo-sensitize the intercalated drug by 488-nm exposure ( Figure 7). As shown in Figure 7A (right panel), this treatment induced local accumulation of the GG-NER damage recognizer XPC-GFP in the photo-sensitized area similarly to local UV-damage infliction (by irradiation through a micro-porous filter) ( Figure 7A, left panel). XPB-GFP exhibited the same behavior as XPC-GFP ( Figure 7B). This indicates that indeed ActD-sensitized DNA is a substrate for XPC binding and the subsequent recruitment of core TFIIH. Remarkably however, TTDA-GFP did not accumulate on this sensitized ActD ( Figure 7C, right panel), whereas it was able to accumulate on a more defined NER-inducing lesion introduced by filter UV irradiation ( Figure 7C, left panel) [ 12]. In addition, also GFP-XPA that accumulates on UV-C (Ultraviolet-C) damaged subnuclear regions ( Figure 7D, left panel) [ 31] was not detectably recruited to photo-sensitized ActD-injured DNA ( Figure 7D, right panel). These data suggest that only NER factors that act early in the sequential assembly process of NER complexes are recruited to these type of lesions, but do not lead to a complete functional complex. Previously, we have found that also the late factor ERCC1-GFP was not immobilized in ActD-treated cells [ 32]. To exclude that ActD per se would specifically reduce the overall mobility of TTDA-GFP, impairing the capability of TTDA to assemble at the sites of DNA damage, we performed strip-FRAP _abc in ActD-treated TTDA-GFP expressing cells ( Figure S3). Although ActD induces partial immobilization of TTDA-GFP (due to transient binding of TFIIH to ActD lesions), the obvious loss of fluorescence at later monitoring time points is indicative for movement of fluorescent molecules over the nuclear membrane. The fact that exchange over the nuclear membrane occurs with the same kinetics as in untreated cells ( Figure S3) suggests that binding to TFIIH is not stabilized in sharp contrast to what was observed with UV light. Apparently, this tethering of TTDA to ActD-bound TFIIH is too short to be visualized as a microscopically visible accumulation. Most importantly, these experiments further showed that increased immobilization of core TFIIH on a non-NER lesion did not change TTDA binding to TFIIH, suggesting that lesions that provoke an abortive NER reaction did not induce a more stable association of TTDA to core TFIIH and exclude the option that simply binding of TFIIH to DNA-induced stabilized anchorage of TTDA.

## Discussion

TFIIH is implicated in a multitude of DNA-transactions, ranging from basal and activated RNA polymerase II transcription, to ribosomal RNA gene expression, participation in GG-NER and TC-NER, and possibly cell-cycle regulation. The complexity of this factor is also reflected by the dynamic composition of distinct subcomplexes, as well as its multiple enzymatic activities: DNA-dependent ATPases and helicases, kinase, and ubiquitin ligase. Participation in multiple reactions complicates the analysis of specific functions when studied in standard in vitro assays. Dynamic regulation of the activity in either one or another process and the existence of different kinetic pools usually escape scrutiny by test tube analysis. Moreover, thermodynamic parameters within mammalian cell nuclei, critical for regulating activity (such as physiologically relevant milieu, high local variations of protein concentrations, and conformation of the DNA substrate), cannot be fully mimicked in vitro. Recent developments in cell biology have opened new opportunities for studying complex processes in the most relevant context, i.e. the living cell.

Prior to in depth live cell analysis of protein dynamics, the biological activity should be carefully checked. In this study we thoroughly analyzed the function of GFP-tagged TTDA. The most pertinent cellular phenotype of TTDA-insufficiency is the NER defect. Remarkably, despite the 4-fold larger size of the tag compared to TTDA itself and the fact that it has to fit in a complex with nine other subunits, that should have numerous interaction partners, TTDA-GFP appeared normally incorporated into TFIIH ( Figure 1C), able to restore normal levels of TFIIH in TTD-A cells ( Figure 1E and 1F), and to correct the UV-sensitivity ( Figure 1B) and RSS after UV irradiation (unpublished data). We conclude that the generated cell line expressing this fluorescently tagged TTDA is a bona fide source to study the actions of this protein.

### Subcellular Localization of TFIIH Subunits

In our live cell studies we have shown that TTDA-GFP and XPD-GFP reside both in the cytoplasm and in the nucleus, contrasting to the strictly nuclear localization of XPB. Photo-bleaching experiments indicated that both proteins do not interact in the cytoplasm. However, application of a newly developed variant of FRAP, FRAP_abc, revealed that TTDA and XPD do interact with TFIIH in the nucleus. In striking contrast to XPB, the association of TTDA to TFIIH (and to a lesser extent also of XPD) is much more dynamic. Previously we found by subcellular fractionation (unpublished data) and others found, by GFP-tagging [ 27], that endogenous XPD is found in both cytoplasm and nucleus. Santagati and colleagues also find p44 to exist in both cytoplasmic and nuclear fractions [ 27] . Thus, it is not unprecedented that some of the TFIIH subunits can exist outside of the context of the complex. Assuming that TTDA is not tightly bound to TFIIH (as our results suggest), a small-sized protein, such as free TTDA (8 kDa for the endogenous and 35 kDa for the tagged version), is expected to exchange between nucleus and cytoplasm through the nuclear pores. Hence a cytoplasmic fraction can be observed.

Both TTDA-GFP and XPD-GFP accumulate in nucleolar foci (previously observed in fixed TTDA-HA-expressing fibroblasts [ 12]). Nucleolar foci co-localize with XPB and p62 in fixed cells and translocate when cells are treated with DRB (unpublished data). This co-localization with the fibrillar nucleolar structure was previously also shown with the other TFIIH component, XPB [ 13, 14].

### TTDA Is Primarily a NER-Specific Factor

Reduced steady-state levels of TFIIH in TTD-A cultured fibroblasts appeared to be a critical determinant in NER efficiency [ 26], but this lower concentration of the crucial basal transcription factor in cultured cells does not seem to largely affect the transcriptional competence. This suggests that NER requires higher concentrations of TFIIH or that the altered structure of the complex mainly affects the NER function rather than the transcription function. Previous live cell kinetic studies [ 14] revealed that a relatively large proportion of the resident TFIIH molecules are recruited to NER sites at which they are bound significantly longer (4–5 min) than when bound for an average transcription initiation event (2–10 s). This difference in kinetic behavior of TFIIH when engaged in either transcription or DNA repair provides an explanation why one process is more sensitive to relative enzyme concentrations than the other. Evaluating the cellular UV-response in TTD-A cells presented an apparent discrepancy between the observed severely reduced levels of DNA repair synthesis (reflecting the rate of NER) and the relatively moderate UV sensitivity [ 9, 26]. Because UV survival reflects a late outcome of UV exposure, whereas DNA repair synthesis provides a snapshot of repair activity, but not it's total impact over time these endpoints are not always directly comparable. A low but long-lasting repair activity (as expected when TFIIH concentrations in TTD-A fibroblasts are low) would still in the end be able to eliminate a substantial fraction of the cytotoxic gene damage, explaining the relatively high UV survival.

The in vitro NER studies [ 29] showed that not only the concentration but also the composition of TFIIH is critical for its function in NER. In the absence of TTDA, only background levels of repair are detected, suggesting that association of TTDA to TFIIH renders this complex more competent for NER at least in vitro. A simple model to explain the NER specificity is that TTDA is required for recruiting subsequent NER factors after the loading of core TFIIH at a lesion. A likely NER-specific role for TTDA could thus be that this subunit specifically interacts with subsequent NER factors. However, considering its very small size and that it interacts already with TFIIH it is hard to imagine that there is much space left for additional interactions. Another possibility is that anchorage of TTDA into TFIIH triggers a conformational change that enables recruitment of other NER factors. This latter alternative may also fit with the observation that lack of TTDA makes TFIIH less stable. Improperly folded proteins are usually vulnerable to degradation. Interaction with TTDA might stabilize the complex either by aiding folding (as a chaperone-like function) or by maintaining tertiary structure. Thus this option would bring reduced repair and instability under the same heading.

### TTDA Is Not Essential for Cell Viability

The complete absence of TTDA in patients, as deduced from the mutation abolishing the start codon, shows that cell viability is not critically depending on TTDA and on high amounts of TFIIH. This is in striking contrast with deletion mutants of each of the previously known TFIIH subunits, which are not viable in yeast and mammals. Incompatibility with live associated with deletions of TFIIH encoding genes was explained by the vital transcriptional role of TFIIH [ 38]. Extrapolation of this hypothesis thus argues that TTDA is not essential for the transcription reaction. Coin and coworkers [ 29] indeed show that in vitro TTDA appeared not to stimulate transcription, whereas is was shown to aid NER. However, part of the non-NER-related features observed in patients with TTD-A (such as the brittle hair) are thought to be derived from an effect on the transcription function [ 39]. This apparent contradiction can be explained by the differential transcriptional program and faith of specific tissues and cell types that are involved in TTD pathology. Previously, it was shown [ 26] that TTDA mutations do not only affect the NER-function but also affect the stability of TFIIH, thereby reducing the steady-state levels of this complex. Decreased stability of TFIIH will mainly affect steady-state levels in cells in which TFIIH de novo synthesis is reduced. Within terminally differentiated cells such as keratinocytes in the hair shaft, a large part of the genome is transcriptionally silent (heterochromatin), except for the final group of abundantly transcribed specialized genes. In these cells, transcription relies on stable TFIIH from earlier differentiation steps. In the absence of TTDA, TFIIH appears unstable, thus this complex may be depleted before the specialized transcription program is finished. Within terminally differentiated keratinocytes, cysteine-rich matrix protein genes are abundantly transcribed. These gene products cross-link keratin filaments and make hair strong. Reduced transcription of this group of genes that are the last to be expressed explains the hallmark feature of TTD, brittle hair. Evidence supporting this model was obtained using a TTD-mutant mouse [ 40]. Further evidence came from studies revealing decreased β-globin expression in precursor erythrocytes (prior to enucleation a large proportion of the genome is transcriptional silent, except the globin genes) causing thalassemia [ 41]. Thus, although TTDA may not to be essential for in vitro transcription [ 29], reduced stability of TFIIH by TTDA mutations will indirectly affect transcription in highly specialized or terminally differentiated cells. The affected transcription function is however compatible with sustaining life, though severely affected. Further analysis, by the future generation of a TTDA mouse-model will help to elucidate the molecular basis for the enigmatic clinical features.

### Model of Dynamic TTDA Regulation

**Figure 8:**
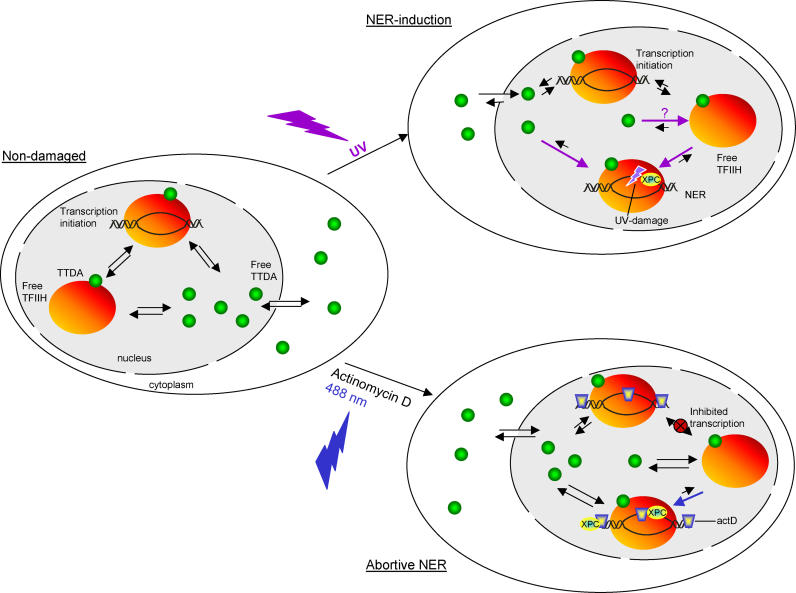
Model of TTDA Binding to TFIIH during Transcription, NER, and Abortive NER Schematic representation of a mammalian cell with the nucleus in gray and nuclear pores simplified by holes in the membrane. TTDA is represented as a green sphere, TFIIH is depicted as an orange ellipse, and XPC is illustrated as a yellow ellipse. Arrows indicate equilibrium (passage through) over the nuclear pores and equilibrium between different TTDA and/or TFIIH molecules. Colored arrows show the changes in the equilibrium after DNA-damage induction or ActD treatment. In “NER-induction” (right, upper), the UV lesion is depicted as a lightning-sign, in “Abortive NER” (right, bottom), ActD is depicted as a blue trapezoid, and the red cross represents the inhibition of transcription.

TTDA can be considered as integral component of TFIIH in biochemical terms ([ 12] and Figure 1C and 1D), however its association to TFIIH in live cells appeared not as firm as some other components. Under normal culture conditions (relatively low presence of genomic injuries) a dynamic equilibrium between TFIIH-associated and free TTDA is present ( Figure 8, schematic cell on the left). In addition, the non-TFIIH bound TTDA molecules can rapidly exchange over the nuclear membrane. Due to its small size this nuclear/cytoplasmic shuttling is likely not an active process but driven by random diffusion. However, exchange over the nuclear membrane is slower than diffusion since it is limited by spatial restrictions (chance of diffusion through a nuclear pore). The fact that a sudden high concentration of DNA lesions by UV-irradiation causes a quick shift in the equilibrium of free versus TFIIH-bound TTDA ( Fig 8, right top cell), suggests that this protein is stronger associated or trapped when the complex is actively engaged in NER. The surprising absence of an effect on TTDA when a non-NER photo-sensitized ActD lesion is introduced is remarkable ( Figure 8, right bottom cell). We showed that early steps of the NER reaction occur on these ‘fake' NER lesions by the efficient loading of the GG-NER initiating factors XPC followed by TFIIH. However, TTDA is not loaded, providing evidence that increased association of TTDA to TFIIH is selective for the presence of NER-specific lesions. Moreover, also subsequent factors as XPA ( Figure 7) and ERCC1 [ 32] were not targeted to these pre-NER complexes. These observations identify TTDA as a primarily NER-dedicated factor, but also provide interesting insight into the NER mechanism. It is tempting to speculate that such lesions trigger the binding of early NER factors, because they induce a DNA conformation resembling a stable helix-distortion and thereby mislead XPC that primarily screens for local distortions. In subsequent steps other aspects of true NER lesions are verified. In the case of photo-sensitized ActD sites, core TFIIH appears to act as a “go or no-go” decision maker in NER. It is possible that the helix-unwinding catalyzed by TFIIH constitutes at least one of the lesion verification steps in normal NER, e.g. when the actual lesion arrests the local unwinding. The extremely high lesion specificity that NER has to achieve (one damage of very wide spectrum of structurally unrelated lesions in more than 10 ^6^ normal nucleotides) cannot be accomplished in one step [ 5]. In the case of ActD intercalation no damage in either of the two strands may be present to stop TFIIH unwinding (or ActD may dissociate when the DNA is opened by TFIIH). This lack of a restriction point may destabilize the early NER complex that will be disassembled.

Taken as a whole, our data show that bona fide NER-lesions selectively trigger an increased stability of TTDA within the TFIIH complex in living cells. This behavior highlights a dynamic, NER-dedicated role of TTDA in relation to lesion-specific function of TFIIH. Our work also underscores the highly versatile and surprisingly diverse nature of TFIIH activities enabling it to participate in a remarkable variety of processes.

## Materials and Methods

### Construction and expression of TTD-A-GFP fusion protein.

Full length TTDA cDNA [ 12] was cloned in-frame into a pEGFP-N1 vector (Clontech, Heidelberg, Germany), at which the first ATG was mutated, to exclude alternative translation start of GFP itself. In addition, full-length human XPD cDNA was cloned in-frame into pEGFP-N2 vector (Clontech). Both constructs were sequenced prior to transfection, using Fugene 6 transfection reagent (Roche, Basel, Switzerland), into respectively TTD-A-deficient human fibroblasts (TTD1BR-Sv) and XP-D-deficient human fibroblasts (XP6BE-Sv).

Stably expressing cells were isolated after subsequent rounds of selection; first Neomycin-dominant marker selection using G418, followed by selection on UV-resistance by exposing them three times to a dose of 6 J/m ^2^ of UV-C light (254 nm) with 2-d intervals. Next, from these mass populations, stably expressing GFP-fusion protein clones were isolated after single cell sorting using the FACSVantage (FACSVantage, Becton Dickinson, Palo Alto, California, United States). Single-cell sorted clones were further analyzed for GFP expression by microscopic evaluation and immunoblot analysis.

### Cell culture and specific treatments.

Cell strains used were SV40-immortalized human fibroblasts: TTD1BR-Sv (TTD-A), with and without stably expressing TTDA-GFP; XPCS2B-Sv (XP-B) stably expressing XPB-GFP [ 14]; XP6BE-Sv (XP-D), with and without stably expressing XPD-GFP; and the NER-proficient cell line (VH10). Cell lines were cultured in a 1:1 mixture of Ham's F10 and DMEM (Gibco, San Diego, California, United States), supplemented with antibiotics and 10% fetal calf serum, at 37 °C, 20% O _2_ and 5% CO _2_.

Treatment with UV-C was performed using a Philips germicidal lamp. A dose of 8 J/m ^2^ was used for total irradiation, and 40 J/m ^2^ for local irradiation, through a micro-porous filter [ 42]. Transcription inhibition was performed by treating the cultured cells with 5,6-dichloro-1-beta-D-ribofuranosylbenzimidazole (DRB, 100 mM) for 3 h. Localized laser-induced damage was achieved by treating the cells with 2 μg/ml of ActD for 1 h. Subsequently, a narrow band across the nucleus was irradiated with 600 pulses of a 488-nm Argon laser at 0.5% laser power (laser power 6.1 A) applied every 100 ms for 1 min. For UV-induced local damage, an isopore polycarbonate filter (Millipore, Billerica, Massachusetts, United States) containing 8-μm diameter pores was used to cover the cells before UV irradiation.

For UV-survival experiments, cells were exposed to different UV-C doses, 2 d after plating. Survival was determined 3 d after UV irradiation by incubation at 37 °C with ^3^H-thymidine, as described previously [ 43].

### Immunoprecipitations and Western blot analysis.

We prepared whole-cell extracts by isolating cells from six petri dishes (14 cm) per cell line. Cells were washed with phosphate-buffered saline (PBS) before lysing them by douncing (20 strokes using a 12.61-mm dounce homogenizer, Bellco Glass) in 2 ml of buffer A (50 mM Tris [pH 7.9], 150 mM NaCl, 20% glycerol, 0.1% Nonidet-P40, and 5 mM β-mercaptoethanol), supplemented with anti-proteases. Cellular extracts were incubated overnight at 4 °C in buffer A with Rabbit polyclonal antibodies to GFP (Abcam, Cambridge, Massachusetts, United States) and mouse monoclonal p44 (1H5), cross-linked to protein A-sepharose beads (Amersham Biosciences, Little Chalfont, United Kingdom). Before immunoprecipitations, cross-linked beads were washed three times with buffer A. After incubation with the extracts, the cross-linked beads were washed extensively with buffer A. Subsequently, beads were boiled in Laemli SDS-PAGE sample buffer, separated on 11% SDS-PAGE, blotted onto nitrocellulose and analyzed using the following antibodies: anti-XPB (1B3) and monoclonal anti-GFP (Roche).

To analyze expression levels of the fusion proteins we prepared whole-cell extracts by sonication. These were separated on an 11% SDS-PAGE and transferred to 0.45 μm nitrocellulose membranes (Millipore). Expression of the fusion proteins was analyzed by hybridizing the membranes with a monoclonal anti-GFP (Roche), followed by a secondary antibody (rabbit anti-mouse) conjugated with horseradish peroxidase (Biosource International, Camarillo, California, United States) and detected using enhanced chemo-luminescence (ECL+ Detection Kit) (Amersham).

### Immunofluorescence assays.

Cells were grown on glass coverslips and fixed with 2% paraformaldehyde at 37 °C. Coverslips were washed with PBS containing 0.1% Triton X-100, three times for 5 min, and subsequently washed with PBS ^+^ (PBS containing 0.15 % glycine and 0.5 % BSA). Cells were incubated at room temperature with primary antibodies (mouse monoclonal anti-XPB, 1:1000, IB3) for 2 h in a moist chamber. Subsequently, coverslips were washed three times with PBS/TritonX-100 and PBS ^+^, incubated 1 h with secondary antibodies, (Cy3-conjugated goat anti-mouse antiserum, Jackson ImmunoResearch Laboratories, Bar Harbor, Maine, United States) at room temperature and again washed three times in PBS/TritonX-100. Samples were embedded in Vectashield mounting medium (Vector Laboratories, Burlingame, California, United States) containing 0.1 mg/ml DAPI (4′-6-diamino-2-phenylindole). Images were obtained by confocal laser scanning microscopy imaging, carried out with a LSM 510 microscope (Zeiss, Oberkochen, Germany).

### FRAP procedures.

3 d prior to microscopy experiments, cells were seeded onto 24-mm-diameter coverslips. Imaging and FRAP were performed on a Zeiss LSM 510 meta confocal laser scanning microscope (Zeiss, Oberkochen, Germany).

FRAP analysis was performed at high time resolution [ 44]. Briefly, a strip spanning the nucleus was photo-bleached for 20 ms at 100% laser intensity (laser current set at 6.1Å). Recovery of fluorescence in the strip was monitored either every 20 ms for 25 s, or every 100 ms for 1 min at 0.5% of laser intensity. Average values from 20 independent measurements were used for every mobility curve. Mobility curves are plotted as relative fluorescence (fluorescence post-bleach divided by fluorescence pre-bleach) measured against time [ 14, 28, 44]. To determine mobility parameters of only TFIIH-bound TTDA-GFP, we developed a novel adaptation of FRAP, designated FRAP_abc. Mobility measurements were obtained after intense bleach pulses (100% laser intensity) were applied to the cytoplasm for about 2 min for TTD-A-GFP and 4 min for XPD-GFP.

To determine residence times of the different TFIIH components at locally damaged areas, we applied FRAP on local damage *.* After local irradiation of TTDA-GFP, XPB-GFP, and XPD-GFP fibroblasts with UV-C light, locally damaged areas were photo-bleached during 3 s with 100% laser intensity. Monitoring of the fluorescence recovery was followed by imaging the cell every 5 s for 200 s. A single image was taken prior to the photo-bleaching. Results are expressed by the ratio between damaged area and undamaged area. The first data point after photo-bleaching is set to 0, while final recovery is normalized to 1. Error bars, in the inset of the curves, represent the standard error of the mean. Statistical significance was checked, whenever two distinct curves were not easily dissociable, by using Student's *t*-test (two-sample, two-tailed) within an appropriate time window: right after the photo-bleaching when evaluating mobility differences or after complete recovery when immobile fractions are being compared.

## Supporting Information

Figure S1 Mobility of TTDA-GFP Mobility of TTDA-GFP in the cytoplasm after bleaching the nuclear pool (red line) compared to the mobility of TTDA-GFP in the cytoplasm without bleaching the nuclear pool (green line). The relative fluorescence (fluorescence post-bleach divided by fluorescence pre-bleach) is plotted against time in seconds.
(193 KB PPT)

Figure S2 Mobility of TTDA-GFP after Knocking Down XPC (A) Scheme of the plasmid used to transfect TTD-A-GFP expressing cells. The construct expresses small-interference RNA against XPC (sequence available upon request), under the control of an H1 (RNA polymerase III) promoter. The plasmid also contains a Mito DsRED_IRES_Hygro transcriptional unit, allowing the selection of transfected clones by the Hygromycin resistance and the mitochondrial DsRED signal.
(B) Example of a TTDA-GFP expressing cell transfected with a mixture of two different XPC small-interference RNA-expressing constructs.
(C) Strip-FRAP_abc of UV-irradiated TTDA-GFP expressing cells knocked-down for XPC (green curve), compared with untreated TTDA-GFP expressing cells (blue curve) and UV-treated TTDA-GFP expressing cells (red curve). Relative fluorescence (fluorescence post-bleach divided by fluorescence pre-bleach) plotted against time in seconds.
(557 KB PPT)

Figure S3 Mobility of TTDA-GFP after ActD Treatment FRAP curve of TTDA-GFP expressing cells untreated (green line) and treated (see Materials and Methods) with ActD (red line). Relative fluorescence (fluorescence post-bleach divided by fluorescence pre-bleach) plotted against time in seconds.
(48 KB PDF)

## Abbreviations

ActD: actinomycin D
FRAP: fluorescence recovery after photo-bleaching
FRAP_abc: FRAP after bleaching the cytoplasm
GFP: green fluorescent protein
GG-NER: global genome nucleotide excision repair
NER: nucleotide excision repair
TC-NER: transcription-coupled nucleotide excision repair
TFIIH: basal transcription factor H
TTD: trichothiodystrophy
TTD-A: trichothiodystrophy group A
TTDA-GFP: green fluorescent protein-tagged TTDA
TTDA-HA: hemagglutinin peptide-tagged TTDA
UV: ultraviolet
UV-C: ultraviolet-C
wt: wild-type
XP: xeroderma pigmentosum
XPA: xeroderma pigmentosum complementation group A protein
XPB: xeroderma pigmentosum complementation group B
XPC: xeroderma pigmentosum complementation group C
XPD: xeroderma pigmentosum complementation group D
