# Supplementary material for: Dynamic Interaction of TTDA with TFIIH Is Stabilized by Nucleotide Excision Repair in Living Cells
Source: PLoS Biol. 2006 May 9;4(6):e156. doi: 10.1371/journal.pbio.0040156 (PMC1457016; doi:10.1371/journal.pbio.0040156)
Supplement: Supplementary file 1 [file pbio.0040156.sg003.pdf]

Figure S3

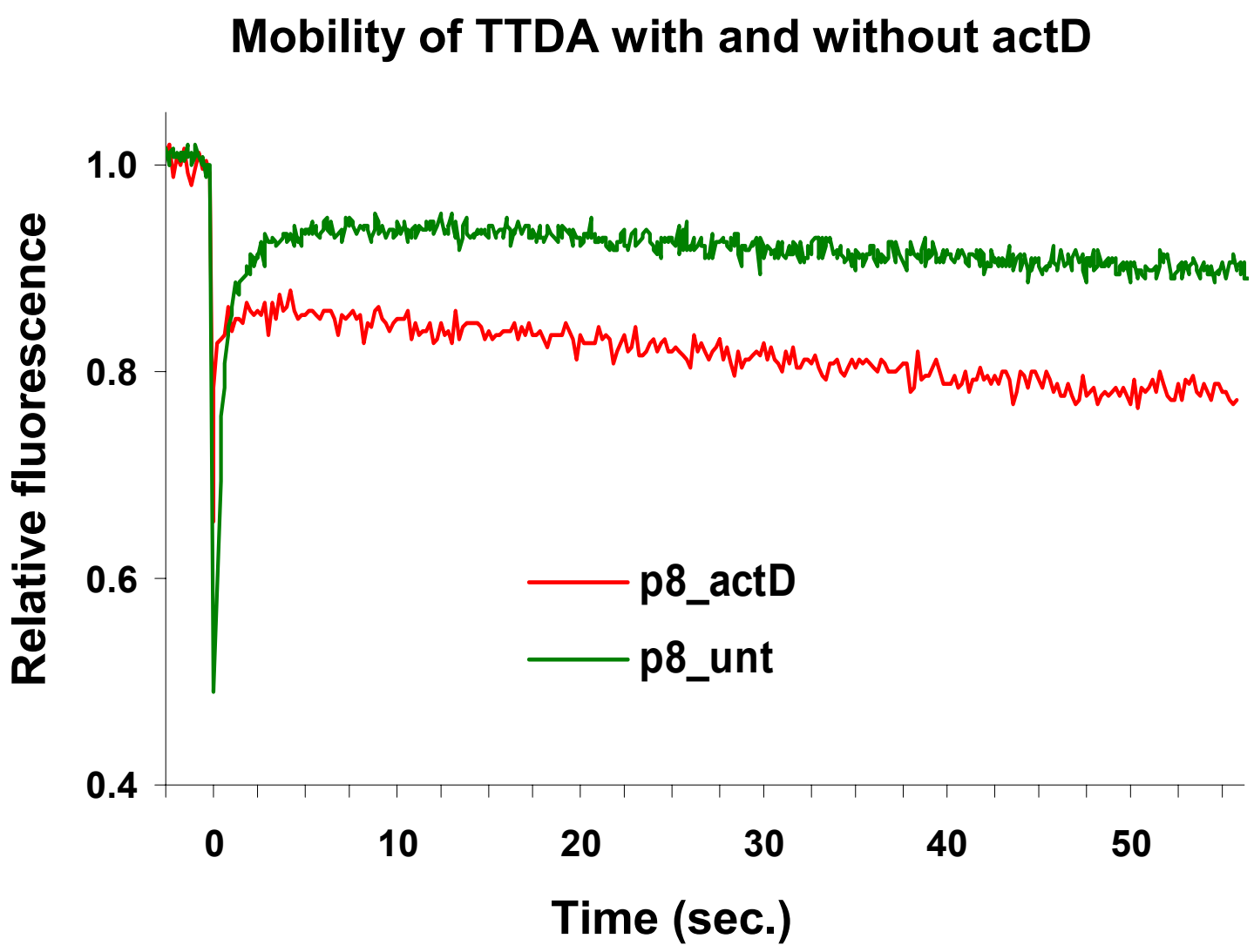
Mobility of TTDA with and without actD
Relative fluorescence
1.0
0.8
0.6
0.4
0
10
20
30
40
50
Time (sec.)
p8_actD
p8_unt
